# Flexible Photodetector with Ultrahigh on/off Current Ratio Based on Monocrystal PbI_2_ Nanosheet via Micro-Spacing In-Air Sublimation

**DOI:** 10.3390/ma19051040

**Published:** 2026-03-09

**Authors:** Chunshuai Yu, Qianqian Du, Yuxing Liu, Yunlong Liu, Wenjun Wang, Shuchao Qin

**Affiliations:** 1 School of Physical Science and Information Engineering, Liaocheng University, Liaocheng 252059, China; 2320110615@stu.lcu.edu.cn (C.Y.); duqianqian@lcu.edu.cn (Q.D.); 2420110517@stu.lcu.edu.cn (Y.L.); liuyunlong@lcu.edu.cn (Y.L.); wjwang@lcu.edu.cn (W.W.); 2 Key Laboratory of Optical Communication Science and Technology of Shandong Province, Liaocheng 252059, China

**Keywords:** lead iodide, flexibility, on/off current ratio, noise level

## Abstract

Two-dimensional (2D) materials are competitive in a diverse range of areas, spanning from electronic and optoelectronic devices to wearable devices, due to their unique physical and chemical characteristics, as well as remarkable flexibility. As a typical 2D material, lead iodide (PbI_2_), featuring a high atomic number and tunable band gap, has been extensively studied in many applications of electroluminescent (EL) devices, photodetectors, and perovskite solar cells. However, high-performance PbI_2_-based photodetectors remain a challenge. Herein, we present a high-performance flexible photodetector based on 2D layered PbI_2_ nanoplates, which were synthesized via a straightforward air sublimation method. The PbI_2_-based photodetector exhibits an excellent photoresponse and the highest responsivity peaks at 34 A/W at 405 nm, together with an ultrahigh transient switching on/off current ratio of 10^7^. Due to a low dark current (10^−14^ A), the device exhibits an extremely low noise level (<10^−26^ A^2^Hz^−1^) and acceptable detectivity (2 × 10^10^ Jones). Furthermore, remarkable mechanical flexibility was observed in the device on a PET substrate, preserving both its electrical conductance and photoresponse stability after 560 bending cycles. Finally, high-resolution imaging applications were implemented under a 100 Hz modulated light signal. This work highlights the superior optoelectrical properties of 2D PbI_2_ growth by the in-air sublimation method and proves its promising future in flexible and wearable optoelectronic devices.

## 1. Introduction

In recent decades, 2D material-based photodetectors have garnered significant attention owing to their exceptional potential for application across intelligent monitoring, as well as flexible electronic equipment, due to their unique structures and superior optoelectronic properties [1,2,3,4,5]. Layered van der Waals materials, such as graphene, transition metal dichalcogenides, and black phosphorus, have been widely studied as the typical 2D materials to fabricate advanced photodetectors. After decades of sustained research and endeavors, remarkable breakthroughs have been achieved in device performance, notably in terms of sensitivity and response speed. However, the performance of these photodetectors based on TMDCs etc. has been unsatisfactory. For instance, although the device achieves an exceptional steady-state on/off current ratio in transfer curves, it demonstrates a poor transient photocurrent switching ratio during time–domain response characterization. An important underlying cause is the typically high defect state density in two-dimensional materials, which governs the carrier recombination dynamics. For practical applications, realizing a genuinely high transient current ratio in a device is of vital importance.

Lead iodide (PbI_2_), which belongs to the series of transition metal halides, is one of the star candidate materials among 2D layered semiconductor materials [6]. The layered nature is characterized by interlayer van der Waals forces, while intralayer bonding primarily consists of Pb-I ionic interactions [7]. Compared with TMDCs, its band structure undergoes a remarkable evolution with increasing dimensionality. For the monolayer state, it has an indirect band gap, while it shifts to a direct band gap when its thickness increases to the bulk state [8]. Up to now, experimental evidence indicates that multilayer PbI_2_ structures exhibit pronounced photoconductivity and photoluminescence [6,7]. In addition, PbI_2_ exhibits great light absorption properties and superior chemical stability, which is of significant importance for advanced optoelectrical devices [8].

Over the past decade, significant research efforts have focused on developing large-scale 2D PbI_2_ nanosheets employing different growth methods [7,9,10,11]. Solution-processing is considered a good technology for obtaining 2D PbI_2_ nanosheets due to its operational simplicity and cost-effectiveness. Although the performance of PbI_2_-based photodetectors is still relatively poor, it may be largely attributed to the solvent residue and moisture absorption [12]. Alternatively, physical vapor deposition (PVD) methods can avoid solvent residue, although this approach generally needs sophisticated equipment, excessively high temperatures, and complicated processes. As is well documented, PbI_2_ tends to undergo phase transition into metastable polymorphs after high-temperature treatment [13,14], which is detrimental for developing high-performance photodetectors. A pressing need exists for the development of a simple new method for growing high-quality 2D PbI_2_, which is essential for addressing the issue of solvent residue. In addition, the potential of flexible photodetection on compliant plastic bases has received only limited investigation, despite its critical importance for emerging wearable and foldable optoelectronic devices.

In this work, high-quality 2D PbI_2_ nanosheets were obtained via a straightforward micro-spacing sublimation technique performed under atmospheric conditions, followed by a comprehensive analysis of their optoelectronic response. X-ray diffraction (XRD) demonstrates its high crystallinity and the 2H phase feature of PbI_2_. The device based on 2D PbI_2_ nanosheets exhibits exceptionally low dark-current levels (10^−14^ A) and noise levels (10^−26^ A^2^Hz^−1^). Under light conditions, the device shows an excellent response, including high responsivity (34 A/W@ 405 nm), an ultrahigh transient switching on/off current ratio of 10^7^, and acceptable detectivity (2 × 10^10^ Jones). Furthermore, remarkable mechanical flexibility was also verified by employing a polyethylene terephthalate (PET) substrate, preserving both its electrical conductance and photoresponse stability after 560 bending cycles. Finally, high-resolution imaging applications were implemented under a 100 Hz modulated light signal. This work highlights the superior optoelectrical properties of 2D PbI_2_ growth by the in-air sublimation method and proves its promising future in flexible and wearable optoelectronic devices.

## 2. Materials and Methods

### 2.1. PbI_2_ Growth and Device Fabrication

Single PbI_2_ crystals were grown via the micro-spacing in-air sublimation (MAS) method under ambient conditions (optimal relative humidity < 40%). A small quantity of powder (approximately 3 mg) was evenly distributed over the bottom substrate (Si/SiO_2_) using a cotton swab. A top growth substrate (Si/SiO_2_) was then placed face-down above the bottom substrate, separated by spacers that defined a sublimation distance of approximately 300 µm. The micro-device was heated on a hot plate from ~296 K to 553 K using a ramp rate of ~65 K·min^−1^ and kept at this temperature for 15 min. After cooling, as-grown PbI_2_ single crystals were obtained on the top substrate. It is noteworthy that a dry environment is crucial for this process; high humidity (e.g., during rainy weather) was found to compromise the stability of the resulting crystals. The upright single crystals were subsequently transferred to a PET substrate by placing them in contact. The device was fabricated by transferring two gold electrodes (approximately 180 nm thick) onto the single crystal under an optical microscope with the assistance of a probe. Traditional electrode fabrication involved hot-evaporating approximately 120 nm thick gold electrodes onto the single crystal using a shadow mask process.

### 2.2. Material Characterization and Device Testing

Optical microscopy observation was conducted on the Zeiss Imager A2m system (Carl Zeiss, Oberkochen, Germany). Raman and absorption spectra were measured using a self-built spectrometer at room temperature and pressure. XRD analysis was conducted using a Panalytical X’Pert3 MRD (Panalytical, Almelo, The Netherlands) instrument that uses a Cu Kα anode. Noise spectrum and transient response measurements were performed using the FS-Pro semiconductor parameter tester (Primarius Technologies Co., Ltd., Shanghai, China). Temperature-dependent tests and real-time responses were recorded using a 6.5K CRX-VF cryogenic vacuum probe station (Lake Shore Cryotronics, Inc., Westerville, OH, USA) in conjunction with a Keithley 4200 semiconductor characterization system (Keithley Instruments, Inc., Cleveland, OH, USA). Photocurrent mapping and imaging were performed with a modulated 405 nm laser in combination with a Keithley 6482 dual-channel picoammeter (Keithley Instruments, Inc., Cleveland, OH, USA) and a Keithley 2400 source meter (Keithley Instruments, Inc., Cleveland, OH, USA). Output and transfer curves were obtained using the same setup, supplemented by a 375/405 nm laser. Incident light power was calibrated and recorded using a Thorlabs PM100D power meter (Thorlabs, Inc., Newton, NJ, USA).

## 3. Results

**Figure 1:**
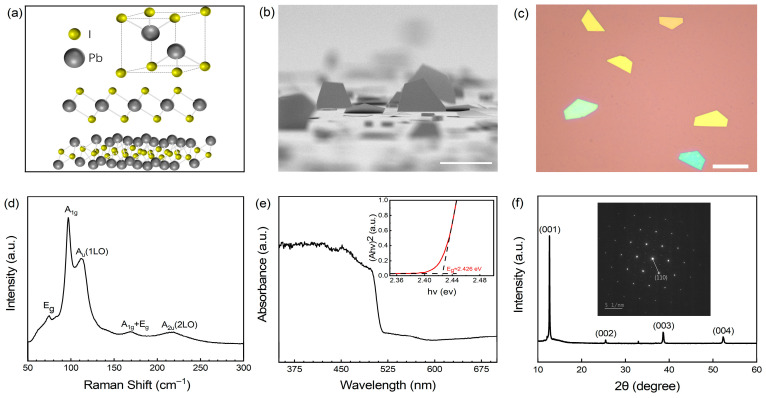
Characterization of the PbI_2_ single crystal. (**a**) Crystalline structure of 2H PbI_2_ single crystal. (**b**) SEM micrograph of the single-crystalline PbI_2_ standing on substrate. Scale bar: 10 μm. (**c**) Optical microscopy observation of the representative PbI_2_ single crystal. Scale bar: 10 μm. (**d**) Raman shift of PbI_2_ crystal. (**e**) UV-Vis absorption spectrum of a PbI_2_ single crystal on quartz. The inset shows the band gap calculated by the absorption of the crystal single. (**f**) XRD and SAED pattern of a PbI_2_ single crystal.

PbI_2_ is a prototypical two-dimensional layered semiconductor. Previous studies have identified more than 30 polymorphs of PbI_2_, with the predominant stacking configurations being 2H, 4H, and 12R [13]. Figure 1a presents a schematic illustration of the most common 2H crystal structure of PbI_2_ under ambient conditions. This structure comprises alternating layers of iodine (I) and lead (Pb) atoms, denoted as I-Pb-I, where two iodine layers encapsulate a single layer of lead atoms. Within each unit layer, the atomic arrangement is stabilized by ionic bonding, whereas the interlayer interactions are primarily governed by van der Waals forces. Under ambient temperature and pressure, this configuration exhibits a hexagonal lattice. The synthesis method employed to obtain PbI_2_ single crystals can be seen in Figure S1 (Supporting Information). Compared with conventional physical vapor-phase or solvent methods, this technique is lower cost, has a simpler operational procedure, and is absent of solvent residues. During our experimental growth process, PbI_2_ nanosheets predominantly exhibited a tendency to stand on the substrate, as illustrated in Figure 1b. Detailed characterization data is shown in Figure S2. This orientation inherently addresses the challenges associated with material transfer and solvent residue in PbI_2_-based systems. Optical microscopy of the sample transferred onto the clean SiO_2_/Si wafer is presented in Figure 1c. As a powerful tool for characterizing lattice arrangement and crystallinity, Raman spectroscopy was first collected under a 532 nm laser (Figure 1d). Here, the strongest *A*_g_ vibrational peak at 96.8 cm^−1^ and dominant *E*_g_ vibrational at 74.9 cm^−1^ belong to layer breathing deformation and shear deformation, indicating a 2H crystal structure with a $D_{3d}^{3}$ space group [15]. Other dominant vibrational peaks at 112.0, 168.5, and 216.3 cm^−1^ correspond to the A_u_(1LO), LA(M) + LO(M), and A_2u_(2LO) vibration modes [16]. The peak positions show no detectable shift relative to their standard values. Additionally, the full width at half maximum (FWHM) of the primary peak is extremely narrow, indicating a high degree of lattice periodicity and long phonon lifetime [17]. Notably, there is no observable peak around 88 cm^−1^, which has been widely reported in the literature as a signature feature originating from stacking faults or defects within the PbI_2_ crystal [18]. These combined spectral features are characteristic of high-quality crystals with minimal defects and strain. To analyze the band gap of the PbI_2_ nanosheet, UV-Vis absorption properties were investigated, as shown in Figure 1e. Using the Tauc Plot method, (*αhν*)^1/2^ *= A*(*hν* − *Eg*), the band gap energy of PbI_2_ nanosheets is estimated to be approximately 2.42 eV, where *α* (absorption coefficient), *h* (Planck’s constant), *ν* (incident photon frequency), *A* (proportionality constant), and *Eg* (semiconductor band gap energy) are used. The value of 2.42 eV falls within the range reported for bulk to monolayer states in previous studies [19].

Here, our PbI_2_ nanosheets were synthesized via the micro-spacing sublimation method at 553 K. Previous studies indicate that PbI_2_ will undergo a phase transition from the 2H to the 12R polytype at approximately 403 K [14], so we performed X-ray diffraction (XRD) measurement to analyze their structural characteristics. As shown in Figure 1f, the sharp diffraction peaks were observed at approximately 12.66°, 25.46°, 38.68°, and 52.32°, which are the typical characteristics of the hexagonal 2H-phase, corresponding to the (001), (002), (003), and (004) crystal planes, in that order. A weaker diffraction peak at 33° may be attributed to the formation of β-PbO, arising from air exposure during the growth process, which is commonly observed in other methods in air [20], but has a negligible impact on device performance (Figure S3). The presence of sharp and symmetrical diffraction peaks signifies a high degree of crystallinity. The highest diffraction peak is observed at 2θ = 12.66°, indicating that the crystals will preferentially grow along the (001) direction. As derived from Bragg’s law (*nλ* = 2*dsin θ*, with *n* = 1 for first-order diffraction, *λ* = 1.54056 Å for Cu Kα radiation, and *d* being the interplanar spacing), we can calculate the interplanar spacing d(001) = 6.98 Å, which corresponds to the unit cell parameter *c* = d(001) of hexagonal 2H-PbI_2_. So, the monolayer thickness of PbI_2_ is approximately 0.7 nm, aligning with previous relevant studies [15]. PbI_2_ typically contains several defects, such as iodine vacancies and interstitial iodine [21]. Here, the narrow full width at half maximum of the diffraction peaks (FWHM = 0.12°) suggests minimal internal stress or lattice defects within the material. In addition to the XRD and Raman results discussed above, transmission electron microscopy (TEM), high-resolution transmission electron microscopy (HRTEM) (Figure S4), and selected-area electron diffraction (SAED) (inset of Figure 1f) measurements provide direct visual evidence of the crystal quality. The well-resolved lattice fringes observed in the HRTEM image and the absence of additional diffraction spots or rings in the SAED pattern further confirm the high crystallinity and low defect state of the PbI_2_ crystals.

**Figure 2:**
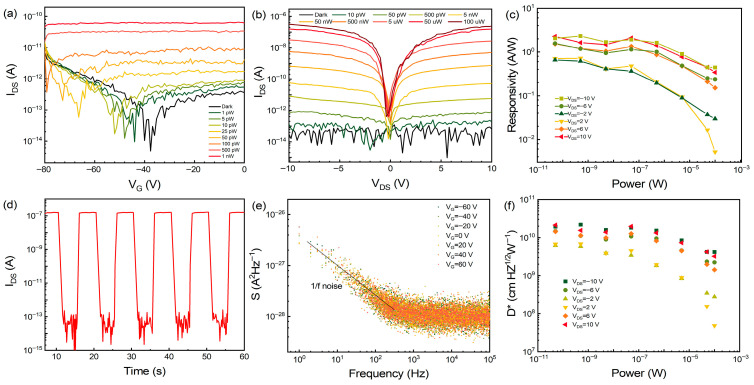
Electrical property of the photodetector. (**a**) Transfer characteristics measured on the device under dark and illumination (405 nm laser) at V_DS_ = −10 V. (**b**) Output curves of PbI_2_ single crystal under different optical powers (V_G_ = 0 V). (**c**) Light power-dependent responsivities under different bias voltages. (**d**) Real-time response of PbI_2_ single crystal device (V_DS_ = 10 V, P = 100 µW). (**e**) Noise current spectra density under different gate voltages. (**f**) Specific detectivity versus incident power at different bias voltages.

With the aim of investigating the optoelectronic properties of PbI_2_ crystals, we fabricated a PbI_2_-based field-effect transistor (FET) on Si/SiO_2_ substrates by transferring Au films on top of the PbI_2_ surface as source and drain electrodes, as illustrated in Figure S5. Figure 2a displays the transfer curves measured on the device at V_DS_ = −10 V. The device exhibits weak n-type conductivity behavior, that is, an intrinsic electron-doping state in dark conditions. Under light illumination (405 nm), the transfer characteristics demonstrate significant photocurrent induction and modulation. With the increase in optical power, the threshold voltage of the minimum current exhibits a negative shift, indicating a typical n-type doping due to a photogating effect [22]. The photoexcited carrier concentration increases the electron concentration in the channel layer, resulting in a large photocurrent. The figure of merit known as responsivity quantifies the efficiency with which a photodetector converts incident optical power into electrical current (*R* = *I_ph_*/*P_in_*). We calculated the responsivity under different gate voltages, and the peak responsivity attains 34 A/W under weak illumination, as depicted in Figure S6. Figure 2b shows the output characteristics recorded from the photodetector at various optical excitation levels at V_G_ = 0 V. Under dark conditions, the source–drain current remains on the order of as low as 10^−14^ A, indicating the promise of an ultra-low noise current. Higher bias voltages drive an increase in photocurrent, which in turn enhances the responsivity at elevated source–drain biases. We utilized the output curve to determine the responsivity (Figure 2c); the maximum responsivity is 2.3 A/W, which is higher than that of most reported PbI_2_-based detectors [10,11,23]. The external quantum efficiency, which quantifies the number of charge carriers collected per incident photon, is determined using the equation *EQE* = *Rhc*/*eλ*, which is about 704%. When the incident light power reaches 100 µW, the current increases to approximately 10^−7^ A, achieving a switching ratio of up to 10^7^. An ultrahigh current switching ratio is significant for requiring high signal-to-noise ratios (SNR). In addition, we recorded the real-time response of the device under the conditions V_DS_ = 10 V and P = 100 µW in Figure 2d. Upon exposure to illumination, the photocurrent rapidly attains its peak current value, and then it can promptly revert to its initial level. The rise time (*t_r_*) is taken as the interval during which the photocurrent rises from 10% to 90% of its saturation level, whereas the decay time (*t_d_*) represents the time for the converse process [23]. The normalized real-time response characteristic at a frequency of 40Hz is shown in Figure S7; the *t_r_*/*t_d_* are approximately 2/3 ms, representing leading-edge performance among PbI_2_-based photodetectors. It is exciting that this high switching ratio can be reproduced in the dynamic response process, which is notoriously difficult for numerous other 2D materials, such as MoS_2_, WS_2_, BP, and so on. Table S1 provides a comparative summary of key photodetection metrics for representative devices based on these materials, further contextualizing the performance of our PbI_2_ photodetector. This excellent performance should be attributed to its high crystallinity and ultra-low defect state concentration. Defect states (such as dislocations, vacancies, and trap states) within the band gap behave as sites where carriers are generated and recombined, significantly increasing the dark current [21,24,25]. Additionally, the low-frequency noise (particularly 1/*f* noise) in photodetectors is predominantly governed by the trapping and de-trapping processes of charge carriers at defect sites [26,27,28]. These stochastic processes cause fluctuations in carrier density and mobility, manifesting as noise in the output current. With an ultra-low trap state density in our high-quality crystals, such carrier fluctuations are effectively suppressed, leading to the observed ultra-low noise performance and ultrahigh switching ratio.

In fields like astronomy and biomedical imaging, where detecting faint light is crucial, the specific detectivity (D*, cm Hz^1^/^2^ W^−1^) becomes a key benchmark. It quantifies a photodetector’s sensitivity to weak radiation by incorporating its signal-to-noise characteristics. In many cases, the specific detectivity is estimated under the assumption that dark-current shot noise dominates, leading to the form $D^{*}=\frac{R(A{)}^{1/2}}{(2eI_{dark}{)}^{1/2}}$, where *I_dark_* is the dark current, and *e* is the elementary charge. Applying this model, our photodetector exhibits a superior D* of 9.7 × 10^12^ Jones in the weak-light regime (50 pW, 405 nm, V_DS_ = 10 V), as summarized in Figure S8. In practice, nevertheless, the noise landscape of a photodetector is more complex. The total noise current typically arises from three sources: shot noise, Johnson noise, and flicker (1/*f*) noise. Their combined contribution is given by $\langle i_{n}^{2}\rangle =[2eI_{dark}+\frac{4k_{B}T}{R_{shunt}}+\langle i_{1/f}^{2}\rangle ]\Delta f$, where *k_B_* is Boltzmann’s constant, and T is the temperature [29]. Experimentally, the noise current could be extracted from the spectral noise density *S*(*f*) via $\langle i_{n}^{2}\rangle =\int_{0}^{\Delta f}S(f)df$. For a narrow bandwidth (~1 Hz), the noise equivalent power (*NEP*) simplifies to $NEP=\frac{\sqrt{\int_{0}^{\Delta f}S(f)df/\Delta f}}{R}\approx \frac{\sqrt{S(f)}}{R}$ [30]. To obtain a reliable D* value that accounts for frequency-varying noise, we measured *S*(*f*) across a range of frequencies (Figure 2e). The spectral noise density follows a 1/*f* trend at low frequencies, indicating that flicker noise—rather than shot noise—dominates the current fluctuations in this regime. From these data, the *NEP* is found to be 0.16 pW/Hz^1^/^2^, which is on par with commercial silicon photodiodes (30 fW/Hz^1^/^2^) [31]. From the measured *NEP*, the specific detectivity D* is determined to exceed 2 × 10^10^ Jones under 405 nm illumination (Figure 2f). Similarly, the response performances for near-ultraviolet irradiation (375 nm) are shown in Figure S9. The responsivity can achieve 1.24 AW^−1^, with an operation speed of *t_r_*/*t_d_* = 3/4 ms and D* = 1× 10^10^ Jones.

**Figure 3:**
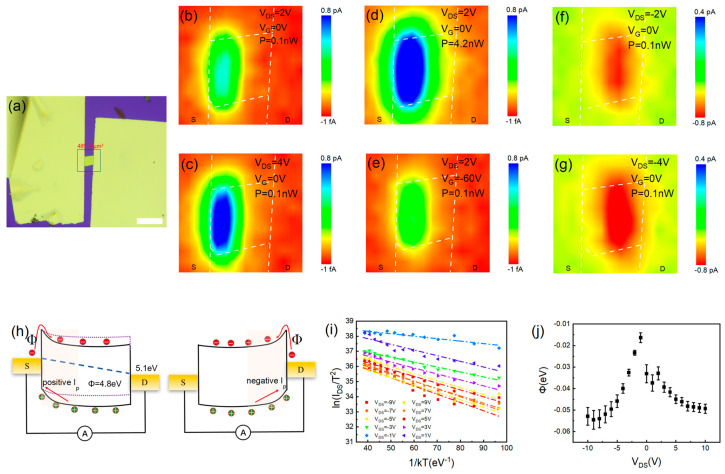
Work principle of photocurrent generation. (**a**) Optical micrograph of the fabricated photodetector. Scale bar: 25 μm. (**b**) Spatial mapping of photocurrent under 405 nm laser signal (V_DS_ = 2 V, V_G_ = 0 V, P = 0.1 nW). (**c**) Photocurrent mapping when bias voltage is increased to 4 V. (**d**) Photocurrent mapping exposed at 4.2 nW. (**e**) Photocurrent mapping under additional conditions (V_DS_ = 2 V, V_G_ = −60 V and P = 0.1 nW). (**f**) Photocurrent mapping (V_DS_ = −2 V, V_G_ = 0 V, P = 0.1 nW). (**g**) Photocurrent mapping (V_DS_ = −4 V, V_G_ = 0 V, P = 0.1 nW). (**h**) Bias-dependent band structure schematics for optoelectronic conversion. (**i**) Arrhenius plot of the device. Linear fits based on the Richardson–Dushman model yield the Schottky barrier. (**j**) Schottky barrier at Au/PbI_2_ interface extracted from Arrhenius plot.

To further elucidate the underlying physical mechanism, we performed high-resolution spatial photocurrent mapping on a representative device using a 405 nm laser, as illustrated in Figure 3a. The photocurrent predominantly emerges in the region adjacent to the PbI_2_ and the electrode (Figure 3b) and increases with applied bias voltage or incident optical power (Figure 3c,d), suggesting a photovoltaic contribution. On account of the work function disparity between PbI_2_ and the Au electrodes, a Schottky barrier forms near the contacts. At this point, the photovoltaic effect is dominant. Applying a negative gate bias, the Schottky barrier would be lower, and the photocurrent would weaken (Figure 3e). Under reverse bias, the dominant photocurrent shifts toward the opposite electrode (Figure 3f,g). As the source–drain bias increases, the governing mechanism transitions toward photogating and photoconductive effects, extending photocurrent generation across the entire channel (Figure 3c,g). To further clarify this behavior, we propose a schematic band diagram model, shown in Figure 3h. The PbI_2_/Au Schottky barrier promotes the dissociation of photoexcited excitons, generating photocurrent primarily near the electrodes. Under low bias, the quasi-flat-band condition within the PbI_2_ channel restricts the dissociation of photogenerated electron–hole pairs, leading to a negligible photocurrent. Increasing the drain bias induces monotonic band bending under the applied electric field, which enhances electron–hole pair separation and extraction and thus yields stronger photocurrent. This bias-dependent evolution of the photocurrent profile is more pronounced under negative bias conditions. To quantify the interfacial barrier height, the photodetector was measured at varying temperatures across 300–105 K. The corresponding Richardson plots are presented in Figure 3i. The data are well described by a linear fit in the Arrhenius representation, confirming that thermionic emission dominates the charge transport across the interface. The thermionic emission process follows the Richardson–Dushman equation: $J=-A^{*}T^{2}\exp\left(\frac{-q\varphi_{B}}{k_{B}T}\right)$, where *q* denotes element charge, and A* is the effective Richardson constant [22]. By analyzing the slope of the *ln*(*I*/*T*^2^) against the *q*/*k_B_T* plot, the barrier height *φ_B_* at the Au/PbI_2_ interface—which reflects the energy barrier for carrier transport—can be determined as a function of the applied bias. The observed difference in *φ_B_* between forward and reverse bias reflects an unintentional asymmetry in the contact barrier, which aligns consistently with the spatial photocurrent mapping results (Figure 3j). Moreover, the increased hole concentration at high drain voltages is likely responsible for the reduction in both interfacial resistance along with effective Schottky barrier height.

**Figure 4:**
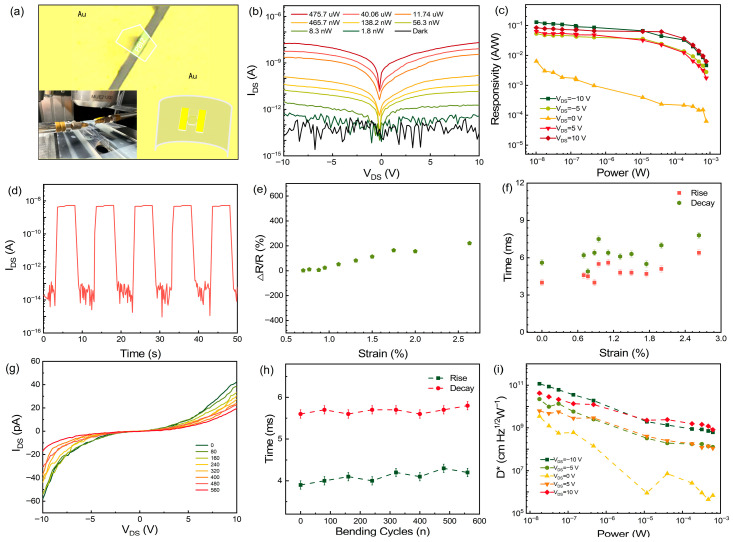
Suitability for the bendable substrate. (**a**) Optical image of PbI_2_ flexible device (PET substrates). Inset shows the photodetector under tensile strain. (**b**) Output curves measured under various light intensities (405 nm). (**c**) Responsivity with respect to incident optical power under different applied biases. (**d**) Real-time response of the device (V_DS_ = 10 V, P = 11.74 µW). (**e**,**f**) Relative change in resistance and response time as a function of the tensile strain. (**g**) Evolution of resistance and response time with applied tensile strain. (**h**) Relative change in response time with the increase in bending cycles. (**i**) Specific detectivity versus incident power at different bias voltages after bending test.

Flexibility is a crucial requirement for next-generation advanced devices to accommodate diverse human life demands. As a representative two-dimensional material, PbI_2_ possesses notable flexibility advantages. Accordingly, we successfully fabricated a simple flexible photodetector using a PET substrate (Figure 4a). Initially, the photoelectric performance of the device on PET was evaluated under 405 nm laser illumination. The output characteristics are shown in Figure 4b, where a clear trend of increasing photocurrent with optical power is observed. Figure 4c presents the responsivity derived from the output curves, which peaks at 0.13 A/W, corresponding to a detectivity of 8.3 × 10^11^ Jones (Figure S10). In general, devices on PET exhibit relatively lower photodetection performance compared with those on SiO_2_/Si (Table S2), which may be attributed to transfer-induced effects and substrate properties. However, this performance remains competitive among previously reported PbI_2_-based transistors. In Figure 4d, the corresponding real-time photoresponse demonstrates that the device can maintain a low dark-current level and a high photocurrent level with a clear switching ratio. Furthermore, the device was evaluated under different tensile strains and repeated bending cycles to examine the variations in resistivity and response time under mechanical deformation (Figure 4e,f). Figure 4e shows a minimal change in resistivity (<25%) when tensile strains are within 1%. However, as the tensile strain increases further, a pronounced increase in resistivity occurs, which can be attributed to the device fabrication process, where the contact between PbI_2_ and the electrodes relies mainly on weak van der Waals forces. As tensile stress escalates, this fragile interface gradually degrades, leading to a rise in contact resistance (Figure S11). Figure 4f displays the measured response times under various tensile strains. Generally, flexible devices exhibit longer response times than rigid ones, primarily due to deep trap states at the PbI_2_/PET interface [32]. Nevertheless, the response speed remains consistent with previously reported values for PbI_2_ field-effect transistors. Given that flexible devices often undergo repeated mechanical deformation, their durability must be thoroughly assessed. Therefore, we performed 560 repeated bending cycles on the device at 0.88% strain. The results show that the device retains excellent output characteristics after bending (Figure 4g), with no significant change in response time (Figure 4h). Further challenges remain regarding increased bending endurance and large-scale uniformity of PbI_2_ crystals fabricated via the micro-spacing sublimation method. Furthermore, we performed light tests under varying laser power densities and evaluated the device’s responsiveness and detectivity by incorporating the light output curve (Figure 4i and Figure S12) after bending cycles. These results underscore the unique advantages of PbI_2_ for future flexible device applications.

**Figure 5:**
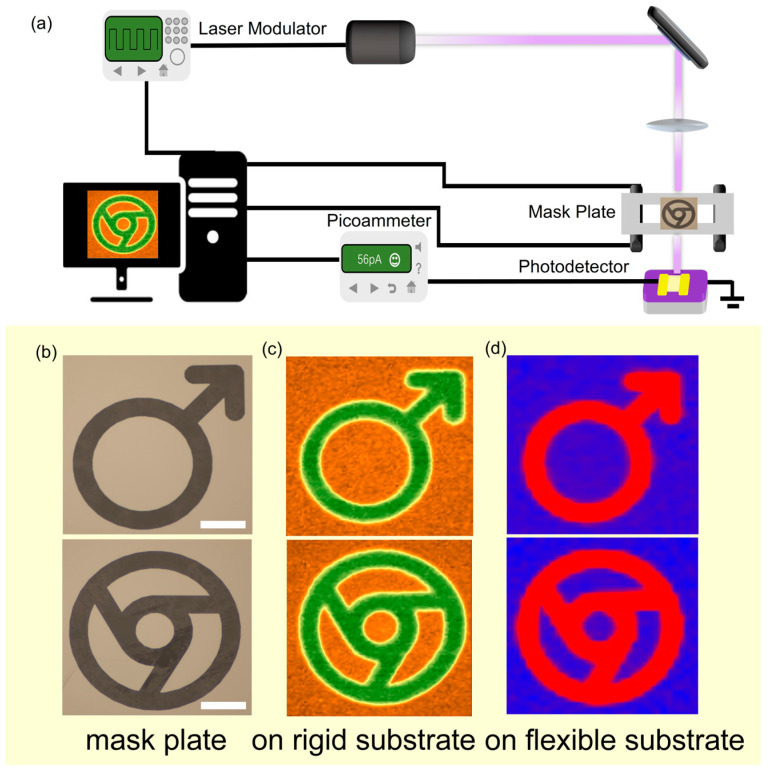
Imaging functionality testing for flexible photodetectors. (**a**) Custom single-pixel imaging test setup schematic. (**b**) View obtained by optical microscopy of the object patterned on the mask plate. Scale bar: 20 μm. (**c**,**d**) Imaging results obtained from the PbI_2_ device on Si/SiO_2_ and PET substrates, respectively.

The imaging capabilities of photodetectors play a critical role in diverse fields such as remote sensing, military applications, and environmental monitoring [33]. To evaluate the imaging performance of PbI_2_ field-effect transistors, we performed a series of imaging tests on devices fabricated on two distinct substrates. Figure 5a presents a schematic diagram of the custom imaging test system developed in our laboratory. A laser operating at a frequency of 10 Hz generates a modulated optical signal, which is then transmitted through a patterned mask plate onto the photodetector. The mask plate is mounted on a controlled piezoelectric platform capable of linear motion in the X–Y plane. By executing a 100 × 100 step scan, the system records the photocurrent variation at each position, ultimately reconstructing a high-resolution image. The shape of the mask plate is illustrated in Figure 5b. Figure 5c,d show the imaging results obtained from PbI_2_ photodetectors on rigid Si/SiO_2_ and flexible PET substrates, respectively. The resulting images exhibit clear resolution and show excellent shape consistency with the photomask pattern. These results demonstrate that the PbI_2_ photodetector possesses excellent imaging capabilities and holds considerable potential for high-resolution imaging applications.

## 4. Conclusions

In conclusion, we successfully synthesized high-quality 2H phase PbI_2_ nanosheets using the micro-spacing sublimation method. The PbI_2_-based device exhibits exceptionally low dark-current levels and noise levels, excellent responsivity (34 A/W@ 405 nm), a fast response speed, and an ultrahigh transient switching on/off current ratio of 10^7^. Additionally, devices fabricated on PET substrates have demonstrated robust mechanical flexibility, maintaining excellent photoelectric performance even after repeated large-angle bending, with minimal degradation in performance. Finally, high-resolution imaging applications were also implemented using a modulated light signal. Our work highlights the feasibility and advantages of the micro-spacing sublimation method for growing PbI_2_ nanosheets and provides such PbI_2_ nanosheets with great promise as an imaging unit in future high-speed optoelectronic systems.

## Data Availability

The original contributions presented in this study are included in the article/Supplementary Material. Further inquiries can be directed to the corresponding author.

## Supplementary Materials

The following supporting information can be downloaded at: https://www.mdpi.com/article/10.3390/ma19051040/s1, Figure S1: growth method of the PbI_2_ single crystal, Figure S2: optical microscopy images of a PbI_2_ single crystal, Figure S3: long-term stability test of the PbI_2_ photodetector, Figure S4: TEM/HRTEM images of a PbI_2_ single crystal, Figure S5: optical microscopy images of PbI_2_ FET, Figure S6: responsivity under different gate voltages of the PbI_2_ device, Figure S7: the normalized real-time response characteristic, Figure S8: detectivity using dark-current-based assumption, Figure S9: response performances for near-ultraviolet irradiation (375 nm), Figure S10: detectivity of the flexible device, Figure S11: optical microscopy images of PbI_2_ flexible FET, Figure S12: the photocurrent response of the flexible device after bending, Table S1: performance comparison of our device with some typical photodetectors, Table S2: performance comparison of rigid with flexible devices. References [34,35,36,37,38,39,40,41,42,43,44,45,46] are cited in the Supplementary Materials.
