# Supplementary material for: Flexible Photodetector with Ultrahigh on/off Current Ratio Based on Monocrystal PbI2 Nanosheet via Micro-Spacing In-Air Sublimation
Source: Materials (Basel). 2026 Mar 9;19(5):1040. doi: 10.3390/ma19051040 (PMC12985976; doi:10.3390/ma19051040)
Supplement: Supplementary file 1 [file materials-19-01040-s001.zip › materials-4149567-supplementary.pdf]

Supporting Information

Flexible Photodetector with Ultrahigh On/Off Current Ratio based on Monocrystal PbI_2 Nanosheet via micro-spacing in-air sublimation

Chunshuai Yu [1,2], Qianqian Du [1,2], Yuxing Liu [1,2], Yunlong Liu [1,2], Wenjun Wang [1,2] and Shuchao Qin [1,2,*]

1 School of Physical Science and Information Engineering, Liaocheng University, Liaocheng 252059, China; 2320110615@stu.lcu.edu.cn (C.Y.); duqianqian@lcu.edu.cn (Q.D.); 2420110517@stu.lcu.edu.cn (Y.L.); liuyunlong@lcu.edu.cn (Y.L.); wjwang@lcu.edu.cn (W.W.)

2 Key Laboratory of Optical Communication Science and Technology of Shandong Province, Liaocheng 252059, China

* Correspondence: qinshuchao@lcu.edu.cn

S1. Growth method of the PbI_2 single crystal

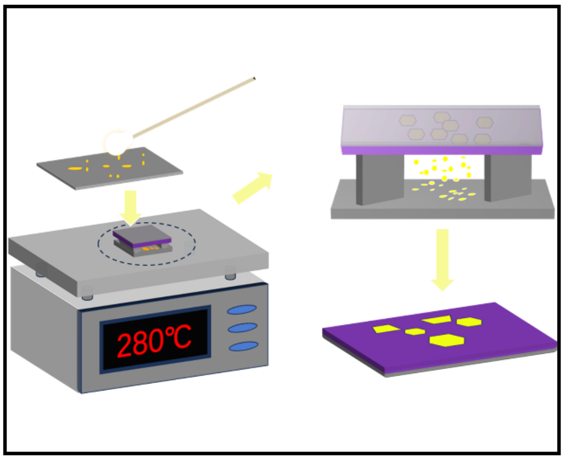

Figure S1. Growth method of the PbI_2 single crystal.

S2. Optical microscopy images of a PbI_2 single crystal

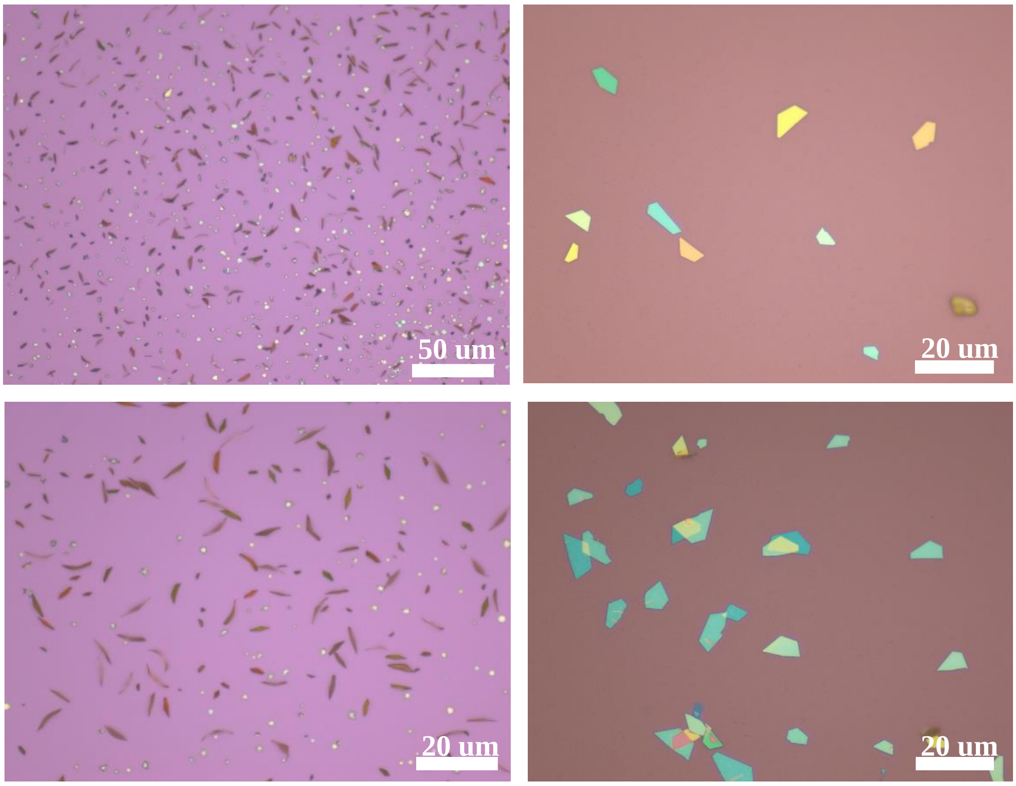

Figure S2. Optical microscopy images of a PbI_2 single crystal.

S3. Long-term stability test of the PbI_2 photodetector

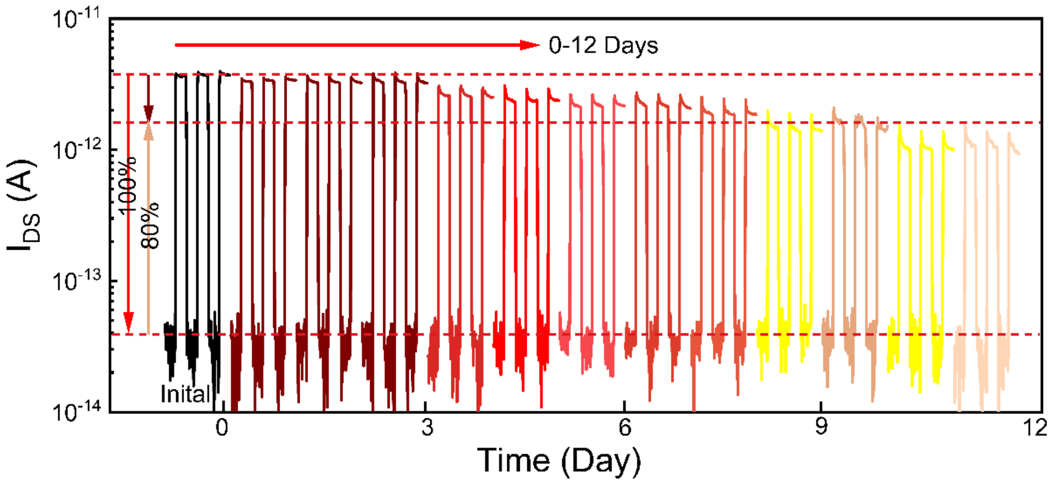

Figure S3. Long-term stability test of the PbI_2 photodetector under ambient conditions. Time-dependent photocurrent measured over 12 days of storage in air (RH 20–40%, ~25 °C). The device retained approximately 80% of its initial photocurrent after 10 days.

S4. TEM/HRTEM images of a PbI_2 single crystal

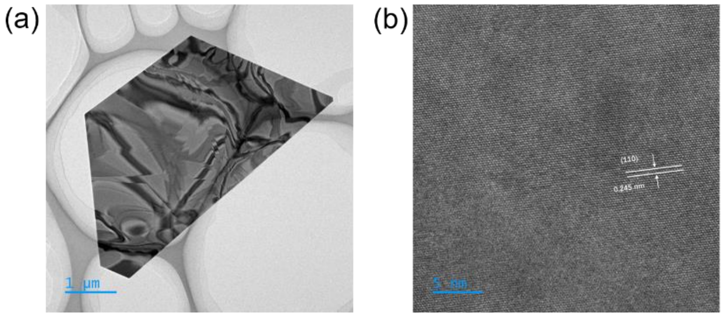

Figure S4. (a) TEM and (b) HRTEM images of a PbI_2 single crystal.

S5. Optical microscopy images of PbI_2 FET

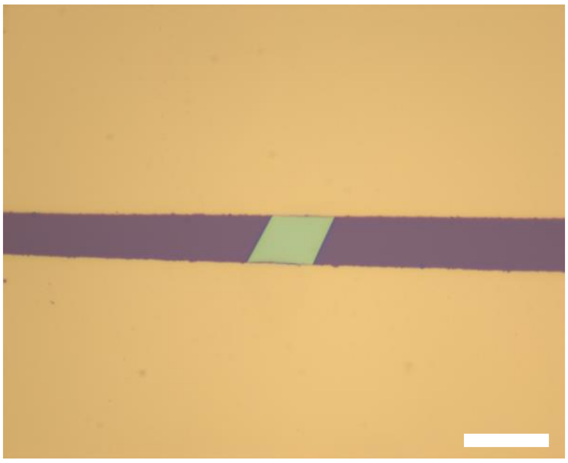

Figure S5. Optical microscopy images of PbI_2 FET. Scale bar: 10 μm.

S6. Responsivity under different gate voltages of the PbI_2 device

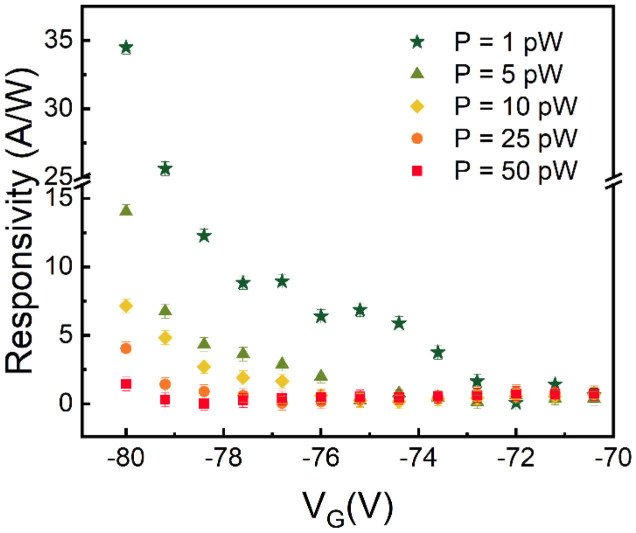

Figure S6. Responsivity under different gate voltages at V_{DS}=10 V of the PbI_2 device.

S7. The normalized real-time response characteristic

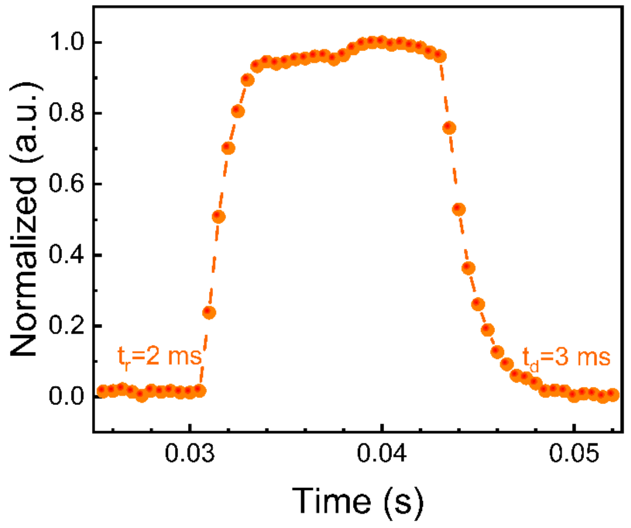

Figure S7. The normalized real-time response characteristic at a frequency of 40Hz under 405nm illumination at V_{DS}=5 V.

S8. Detectivity using dark current-based assumption

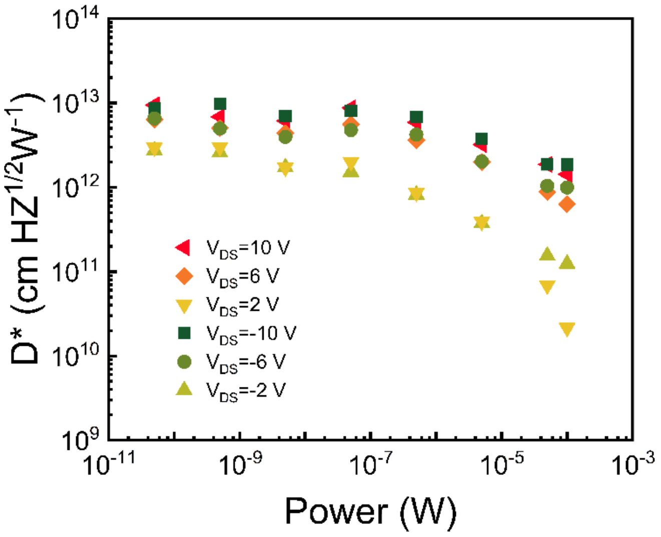

Figure S8. Detectivity using dark current-based assumption. An ultrahigh specific detectivity of 9.7×10^{12} Jones is obtained under weak light (50 pW, 405 nm) at V_{DS}=10V.

S9. Response performances for near-ultraviolet irradiation (375 nm)

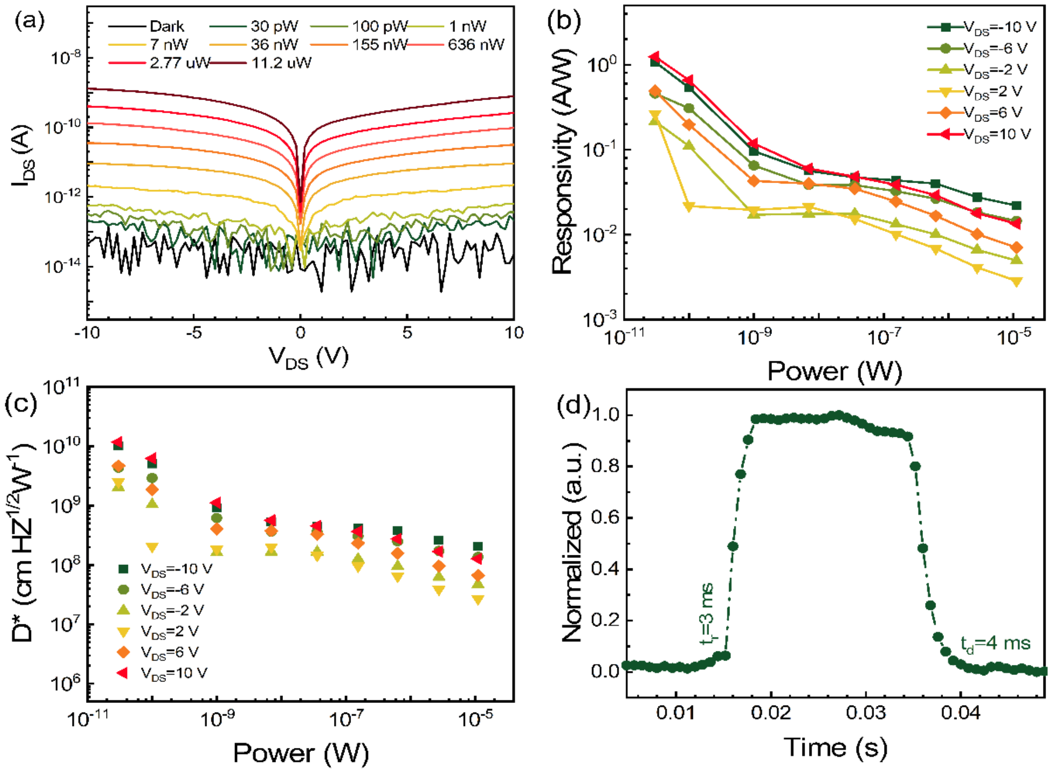

Figure S9. Response performances for near-ultraviolet irradiation (375 nm). (a) The output curves on light conditions. (b)-(c) Responsivity and Specific detectivity calculated from the output curves. (d) Rise and decay time of the photocurrent response in a single cycle.

S10. Detectivity of the flexible device

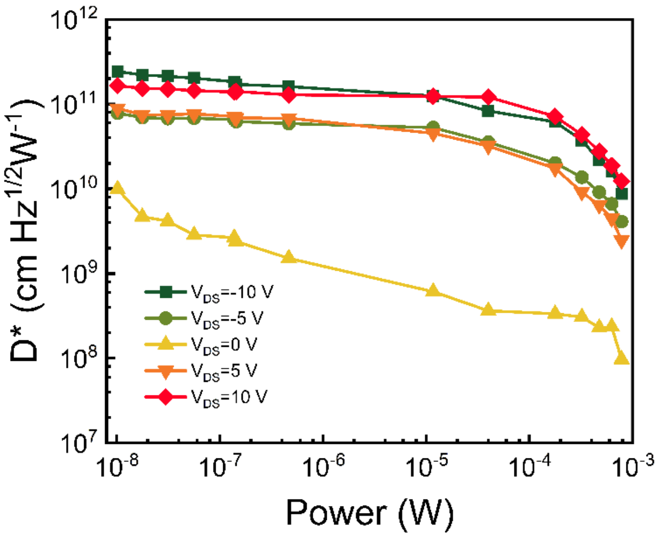

Figure S10. Detectivity of the flexible device at 1.8 nW laser power (405nm laser) derived from the output curves.

S11. Optical microscopy images of PbI_2 flexible FET

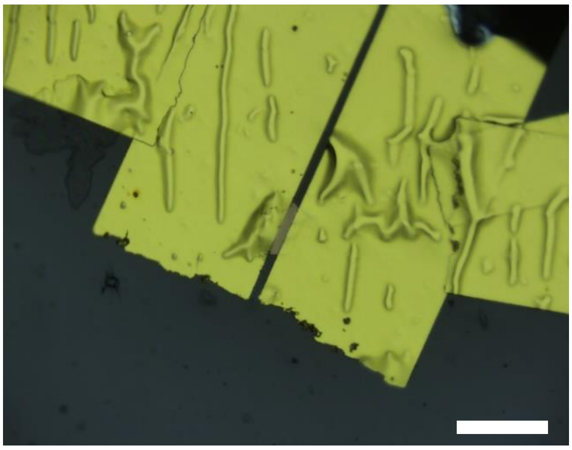

Figure S11. Optical microscopy images of PbI_2 flexible FET. As tensile stress escalates, the fragile interface gradually degrades, leading to a rise in contact resistance. Scale bar: 10 μm.

S12. The photocurrent response of the flexible device after bending

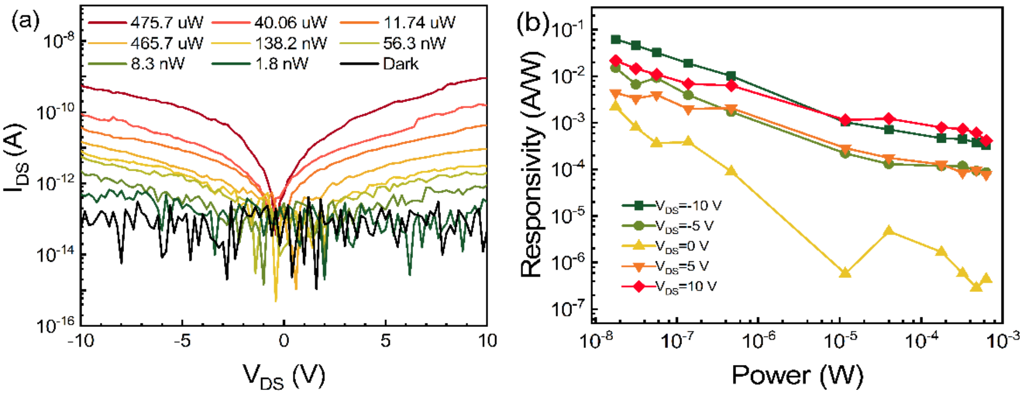

Figure S12. The photocurrent response of the device after bending cycles at 0.88% strain. (a) Output curves of device under various light intensities (405nm laser). (b) Responsivity as a function of the light power.

Table S1. Performance comparison of our device with some typical photodetectors

Materials	R (A/W)	D^* (Jones)	Response Time (ms)	Growth Methods	Dark Current(A)	Light On/off	Ref
PbI_2	3.4	$2*10^{10}$	2/3	MAS	$1*10^{-14}$	10^7	Our work
PbI_2	0.04	$2.56*10^{11}$	18/22	PVD	$1*10^{-11}$	10^2	[1]
PbI_2	0.41	$3.1*10^{11}$	86/150	PVD	$5*10^{-12}$	NA	[2]
PbI_2	0.65	$9.5*10^{12}$	2/3	Solution	$2*10^{-12}$	10^6	[3]
PbI_2	0.51	$4*10^{10}$	14/31	Solution	$1*10^9$	63	[4]
PbI_2	0.013	$3*10^{10}$	425/41	CVD	$4*10^{-12}$	10^3	[5]
MoS_2	33	$6.1*10^{11}$	38/190	Mechanical Exfoliation	NA	10^6	[6]
1T/2H MoS_2	0.089	$4.98*10^{11}$	6.2/5	Electrochemical Intercalation	NA	700	[7]
$MnPS_3/MoS_2$	0.153	$1.61*10^{11}$	0.87/2.56	Mechanical Exfoliation	NA	$3.3*10^5$	[8]
WS_2	0.018	NA	<4.5	CVD	NA	10^6	[9]
$GeAs/WS_2$	0.509	$1.08*10^{12}$	NA	Mechanical Exfoliation	NA	10^3	[10]
Graphene/BP	0.183	$6.689*10^8$	0.012/0.013	Mechanical Exfoliation	NA	NA	[11]
$BP/MoSe_2$	0.0032	$1.4*10^9$	NA	Mechanical Exfoliation	NA	10	[12]
BP/InSe	53.8	NA	35/48	Mechanical Exfoliation	$1*10^{-6}$	NA	[13]
1D/2D WSe_2	0.0346	$3.24*10^9$	23.1/16.8	CVD	NA	$1.5*10^3$	[14]
WSe_2/In_2Se_3	0.026	NA	2.22/2.38	Mechanical Exfoliation	$1*10^{-13}$	$1.2*10^5$	[15]
$MoTe_2/MoSe_2$	1.5	$2.7*10^{12}$	<30/<35	Mechanical Exfoliation	$1*10^{-13}$	10^4	[16]
$PtSe_2/Ge$	0.64	$1.82*10^{12}$	0.0067/0.015	Post-Selenization	NA	$1.5*10^4$	[17]

Table S2. Performance comparison of rigid with flexible devices.

Substrates	Dark current (A)	R (A/W)	D^* (Jones)	t_r/t_d (ms)	On/off ratio
Si/Sio_2	~10^{-14}	2.3	$9.7*10^{12}$	2/3	10^7
PET	~10^{-14}	0.13	$8.3*10^{11}$	3.8/5.2	10^5

Reference

1. Zhong, M.; Zhang, S.; Huang, L.; You, J.; Wei, Z.; Liu, X.; Li, J. Large-Scale 2D PbI_2 Monolayers: Experimental Realization and Their Indirect Band-Gap Related Properties. *Nanoscale* **2017**, *9*, 3736–3741, doi:10.1039/C6NR07924E.
2. Lan, C.; Dong, R.; Zhou, Z.; Shu, L.; Li, D.; Yip, S.; Ho, J.C. Photodetectors: Large-Scale Synthesis of Freestanding Layer-Structured PbI_2 and $MAPbI_3$ Nanosheets for High-Performance Photodetection (Adv. Mater. 39/2017). *Advanced Materials* **2017**, *29*, adma.201770284, doi:10.1002/adma.201770284.
3. Liu, D.; Chen, R.; Liu, F.; Zhang, J.; Zhuang, X.; Yin, Y.; Wang, M.; Sa, Z.; Wang, P.; Sun, L.; et al. Flexible Omnidirectional Self-Powered Photodetectors Enabled by Solution-Processed Two-Dimensional Layered PbI_2 Nanoplates. *ACS Appl. Mater. Interfaces* **2022**, *14*, 46748–46755, doi:10.1021/acsami.2c13373.
4. Xiao, H.; Liang, T.; Xu, M. Growth of Ultraflat PbI_2 Nanoflakes by Solvent Evaporation Suppression for High-Performance UV Photodetectors. *Small* **2019**, *15*, 1901767, doi:10.1002/smll.201901767.
5. Pallares, R.M.; Su, X.; Lim, S.H.; Thanh, N.T.K. Fine-Tuning of Gold Nanorod Dimensions and Plasmonic Properties Using the Hofmeister Effects. *J. Mater. Chem. C* **2016**, *4*, 53–61, doi:10.1039/C5TC02426A.
6. Chen, X.-Y.; Su, D.; Li, K.-H.; Song, Y.-J.; Xia, P.; Zhang, X.-Y. Multilayer MoS_2 Photodetector with Broad Spectral Range and Multiband Response. *Adv Devices Instrum* **2024**, *5*, 0042, doi:10.34133/adi.0042.
7. Tang, D.; Liu, Y.; Zheng, X.; Lu, Y.; Xie, Y.; Wang, G.; Liu, W.; Gu, C.; Shen, X. High Performance Self-Powered and Broadband Photodetector Based on 1T/2H MoS_2 Schottky Homojunction. *Optical Materials* **2025**, *163*, 116955, doi:10.1016/j.optmat.2025.116955.
8. Liang, C.; Nan, H.; Shao, K.; Wang, C.; Weng, Z.; Wu, Z.; Jian, J.; Xiao, S.; Gu, X. Ultrasensitive MoS_2 Photodetectors Enabled by $MnPS_2$ Interface Engineering. *Materials Science in Semiconductor Processing* **2026**, *202*, 110153, doi:10.1016/j.mssp.2025.110153.
9. Lan, C.; Li, C.; Yin, Y.; Liu, Y. Large-Area Synthesis of Monolayer WS_2 and Its Ambient-Sensitive Photo-Detecting Performance. *Nanoscale* **2015**, *7*, 5974–5980, doi:10.1039/C5NR01205H.
10. Xiong, J.; Li, J. Multifunctional GeAs/WS2 Heterojunctions for Highly Polarization-Sensitive Photodetectors in Short-Wave Infrared Range.
11. Liu, C.; Guo, J.; Yu, L.; Li, J.; Zhang, M.; Li, H.; Shi, Y.; Dai, D. Silicon/2D-Material Photodetectors: From near-Infrared to Mid-Infrared. *Light Sci. Appl.* **2021**, *10*, 123, doi:10.1038/s41377-021-00551-4.
12. Abderrahmane, A.; Woo, C.; Ko, P.J. Black Phosphorus/Molybdenum Diselenide Heterojunction-Based Photodetector. *J. Electron. Mater.* **2021**, *50*, 5713–5720, doi:10.1007/s11664-021-09097-y.
13. Cao, R.; Wang, H.; Guo, Z.; Sang, D.K.; Zhang, L.; Xiao, Q.; Zhang, Y.; Fan, D.; Li, J.; Zhang, H. Black Phosphorous/Indium Selenide Photoconductive Detector for Visible and Near-Infrared Light with High Sensitivity. *Advanced Optical Materials* **2019**, *7*, 1900020, doi:10.1002/adom.201900020.
14. Zhang, B.; Ao, Z.; Lan, X.; Zhong, J.; Zhang, F.; Zhang, S.; Wang, L.; Chen, P.; Wang, G.; Yang, X.; et al. Self-Rolled-up WSe_2 1D/2D Homojunctions: Enabling High Performance

Self-Powered Polarization-Sensitive Photodetectors.
15. Liu, B.; Tang, B.; Lv, F.; Zeng, Y.; Liao, J.; Wang, S.; Chen, Q. Photodetector Based on Heterostructure of Two-Dimensional WSe_2 /In_2Se_3. *Nanotechnology* **2020**, *31*, 065203, doi:10.1088/1361-6528/ab519b.
16. Luo, H.; Wang, B.; Wang, E.; Wang, X.; Sun, Y.; Liu, K. High-Responsivity Photovoltaic Photodetectors Based on $MoTe_2$/$MoSe_2$ van Der Waals Heterojunctions. *Crystals* **2019**, *9*, 315, doi:10.3390/cryst9060315.
17. Li, X.; Wu, S.; Wu, D.; Zhao, T.; Lin, P.; Shi, Z.; Tian, Y.; Li, X.; Zeng, L.; Yu, X. In Situ Construction of PTSE2/Ge Schottky Junction Array with Interface Passivation for Broadband Infrared Photodetection and Imaging. *InfoMat* **2024**, *6*, e12499, doi:10.1002/inf2.12499.
